# Hybrid Graphene-Silicon Based Polarization-Insensitive Electro-Absorption Modulator with High-Modulation Efficiency and Ultra-Broad Bandwidth

**DOI:** 10.3390/nano9020157

**Published:** 2019-01-27

**Authors:** Yin Xu, Feng Li, Zhe Kang, Dongmei Huang, Xianting Zhang, Hwa-Yaw Tam, P. K. A. Wai

**Affiliations:** 1 Photonics Research Centre, Department of Electrical Engineering, The Hong Kong Polytechnic University, Hung Hom, Hong Kong; xuyinseu@gmail.com (Y.X.); eehytam@polyu.edu.hk (H.-Y.T.); 2 The Hong Kong Polytechnic University Shenzhen Research Institute, Shenzhen 518057, China; 3 Photonics Research Centre, Department of Electronic and Information Engineering, The Hong Kong Polytechnic University, Hung Hom, Hong Kong; zhe.kang@polyu.edu.hk (Z.K.); 17901678r@connect.polyu.hk (D.H.); buptxtzhang@gmail.com (X.Z.); alex.wai@polyu.edu.hk (P.K.A.W.)

**Keywords:** silicon photonics, integrated optical devices, electro-optic modulator, graphene

## Abstract

Polarization-insensitive modulation, i.e., overcoming the limit of conventional modulators operating under only a single-polarization state, is desirable for high-capacity on-chip optical interconnects. Here, we propose a hybrid graphene-silicon-based polarization-insensitive electro-absorption modulator (EAM) with high-modulation efficiency and ultra-broad bandwidth. The hybrid graphene-silicon waveguide is formed by leveraging multi-deposited and multi-transferred methods to enable light interaction with graphene layers in its intense field distribution region instead of the commonly used weak cladding region, thus resulting in enhanced light–graphene interaction. By optimizing the dimensions of all hybrid graphene-silicon waveguide layers, polarization-insensitive modulation is achieved with a modulation efficiency (ME) of ~1.11 dB/µm for both polarizations (ME discrepancy < 0.006 dB/µm), which outperforms that of previous reports. Based on this excellent modulation performance, we designed a hybrid graphene-silicon-based EAM with a length of only 20 µm. The modulation depth (MD) and insertion loss obtained were higher than 22 dB and lower than 0.23 dB at 1.55 µm, respectively, for both polarizations. Meanwhile, its allowable bandwidth can exceed 300 nm by keeping MD more than 20 dB and MD discrepancy less than 2 dB, simultaneously, and its electrical properties were also analyzed. Therefore, the proposed device can be applied in on-chip optical interconnects.

## 1. Introduction

Silicon photonics based on the mature silicon-on-insulator (SOI) platform has aroused tremendous interest for building compact, high performance, and energy-efficient photonic integrated circuits (PICs), which has also promoted the rapid development of optical interconnects, especially for data center and telecom applications [1,2,3,4]. To keep up with the ever-increasing demand of high capacity on-chip optical interconnects, much attention has been focused on the development of advanced multiplexing technologies (e.g., wavelength-division-multiplexing [5], polarization-division-multiplexing [6], mode-division-multiplexing [7]) and their hybrid multiplexing types [8]. However, these multiplexing technologies will inevitably impose higher performance requirements on optical modulators, e.g., higher modulation efficiency (ME), larger optical bandwidth, ultra-fast modulation speed, ultracompact size, lower energy consumption, and complementary metal-oxide-semiconductor (CMOS) processing compatibility [1]. These requirements cannot be well satisfied by silicon modulators which are based on weak plasma dispersion effects [9]. As a result, new materials, structures, and mechanisms have been introduced to the SOI platform to improve the modulation performance of silicon modulators. For instance, III–V epitaxial materials were transferred to an SOI wafer forming a hybrid silicon electro-absorption modulator (EAM) [10], which achieves a high ME of 0.25 dB/µm and over 67 GHz modulation bandwidth at the wavelength of 1.3 µm, while at the cost of incompatible CMOS processes and complex fabrication procedures. The silicon-organic hybrid (SOH) platform—filling nano-slot waveguides with organic cladding materials—was developed, and the corresponding SOH-based electro-optic modulator consumes only 0.7 fJ/bit for the device length of 1 mm with 12.5 GHz modulation bandwidth [11]. The effort to decrease the device footprint is likely to be in progress. Other waveguide structures could also be applied onto this platform, e.g., slot photonic crystal waveguide [12]. Moreover, by means of the large thermo-optic coefficient of silicon [13], the thermal modulation method has also been explored for silicon modulators, but the modulation speed was limited and the energy consumption was relatively high. Therefore, new hybrid silicon modulators with better performance and smaller size are highly desirable to fulfill the modulation requirements of high capacity on-chip optical interconnects [14].

Recently, graphene, a single-atom-layer of graphite with its atoms arranged in a hexagonal lattice [15], has become a promising two-dimensional (2D) material in the field of photonics and electronics with its exceptional optical/electrical properties such as ultra-high carrier mobility (e.g., >200,000 cm^2^·V^−1^·s^−1^ at room temperature) [16], tunable conductivity, broad bandwidth, zero bandgap, and CMOS compatible processes [17,18,19]. These remarkable properties match up well with the performance requirements and developing directions of silicon modulators if graphene can be effectively integrated on the SOI platform [20,21]. In 2011, the first single-layer graphene-on-silicon EAM was reported with an ME of 0.1 dB/µm and modulation bandwidth of 1.2 GHz [22]. In 2012, a double-layer version of graphene-on-silicon EAM was also developed with an ME of ~0.16 dB/µm and modulation bandwidth of 1 GHz [23]. Notwithstanding, the reported ME are relatively low because of the ultra-thin thickness of graphene (~0.34 nm) and the weak evanescent wave in the cladding regions of silicon waveguide interaction with the graphene layers coated, resulting in reduced light–graphene interaction (LGI). In order to effectively improve LGI, special waveguide structures with enhanced light intensities have been exploited to combine with graphene flake, such as slot waveguides [24], surface plasmon polariton waveguides [25], and hybrid plasmonic waveguides [26], instead of the commonly used rid/ridge waveguides. The device ME is significantly increased (e.g., 0.84 dB/µm) [27], thus the device length can be shortened and the energy consumption reduced. However, these devices are intrinsically polarization-dependent, particularly for some strong light-confinement structures, operating under only a single-polarization state. As a result, these devices would not satisfy the requirements of dual-polarization operation for the polarization-division-multiplexing [6] and multi-dimensional hybrid multiplexing [8] systems. It is therefore necessary to develop polarization-independent or polarization-insensitive high performance graphene-based optical modulators. By transferring graphene layers on an isosceles trapezoid silicon nanowire and capping with another silicon nanowire, a polarization-independent EAM has been proposed, with an extinction ratio (ER) higher than 20 dB in a length of 30 µm [28]. However, its ER discrepancy for the two polarizations is high (~2.3 dB) and the precise control of the slanted angle of the silicon nanowire might pose challenges for practical fabrication processes. Furthermore, based on a typical double-layer graphene-on-silicon structure [23], polarization-insensitive modulation can also be realized by optimizing the dimensions of the bottom silicon nanowire, while its obtained performance is limited with an ME of only 0.29 dB/µm and a transmission loss of ~2 dB [29]. Therefore, in order to effectively enhance the performance of graphene-based polarization-insensitive optical modulators, new structures and operating mechanisms remain to be developed.

In this paper, we propose a highly-efficient, ultra-broadband, and polarization-insensitive EAM based on a multi-deposited and multi-transferred hybrid graphene-silicon waveguide structure. The input and output waveguides are conventional silicon nanowires. In order to enhance the ME or LGI for both polarizations, the proposed hybrid graphene-silicon waveguide was designed such that the light interaction with graphene layers happens in the intense field distribution region rather than in the cladding region with weak evanescent waves as in previously reported work. Meanwhile, hexagonal boron nitride (hBN) was used as the spacer or insulating layer to encapsulate graphene layers and maintain the high carrier mobility of graphene [30]. By optimizing the dimensions of all hybrid graphene-silicon waveguide layers, polarization-insensitive modulation was realized with an ME of ~1.11 dB/µm and an ME discrepancy of lower than 0.006 dB/µm for both polarizations, which are better than previous reports [28,29]. We then propose a hybrid graphene-silicon-based polarization-insensitive EAM with a modulation length of 20 µm. The modulation depth (MD) and insertion loss (IL) were higher than 22 dB and lower than 0.23 dB, respectively, at 1.55 µm. The corresponding available bandwidth reached over 300 nm (from 1.367 to 1.668 µm, covering the whole S to L bands and most of the E and U bands) with a MD greater than 20 dB and MD discrepancy less than 2 dB, simultaneously. We also study the electrical properties such as 3 dB modulation bandwidth and energy consumption. Finally, we discuss some feasible ways to further enhance the device performance. The rest of this paper is organized as follows: Section 2 presents the device design and operating principle of the proposed hybrid graphene-silicon based EAM; Section 3 analyzes the optical modulation properties of the hybrid graphene-silicon waveguide structure; Section 4 gives the numerical results and discusses the proposed device; and the conclusions are drawn in Section 5.

## 2. Device Structure and Principle

**Figure 1:**
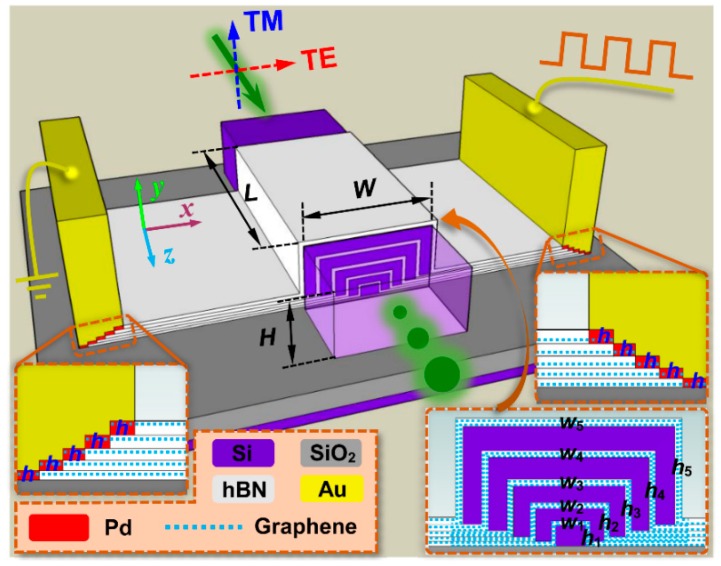
Schematic of the proposed hybrid graphene-silicon-based polarization-insensitive electro-absorption modulator (EAM). The insets illustrate the enlarged cross-sectional views of the central hybrid graphene-silicon waveguide and the metal contacts on both sides. *w_m_* and *h_m_*, where *m* = 1, 2, …, 5, represent the width and thickness, respectively, of the five inverted U-shaped graphene-silicon waveguide layers from inside to outside. *W*, *H*, and *L* represent the width, thickness, and length of the proposed EAM, respectively.

Figure 1 shows the three-dimensional (3D) schematic of the proposed hybrid graphene-silicon-based polarization-insensitive EAM. The insets are the enlarged cross-sectional views of the central hybrid graphene-silicon waveguide and the metal contacts on both of its sides. Two graphene layers grown by the way of chemical vapor deposition (CVD) were transferred to cover the surface of the bottom silicon nanowire for every inverted U-shaped graphene-silicon layer. The corresponding five graphene-silicon layers with optimized dimensions were successively deposited and transferred onto the buried oxide layer of a SOI wafer to form the proposed device. Within this structure, hBN was employed as the insulating layer between the two graphene layers, forming a typical capacitor structure. If this capacitor is applied by bias voltage, the accumulation of free carriers will be formed on one graphene layer and the elimination of those from another one accordingly, leading to an effective tuning of the Fermi level of both graphene layers and thus generating an electro-absorption (EA) modulation effect [31]. The widths and thicknesses of the five inverted U-shaped graphene-silicon layers are *w_m_* and *h_m_*, where *m* = 1, 2, …, 5 from inside to outside, respectively, and the spacer layer thickness of every graphene-silicon layer is *h* = 10 nm, as illustrated in Figure 1. By optimizing these parameters, the light field will strongly interact with the graphene layers in its intense distribution region for both polarizations, which is in the interior rather than the exterior of the hybrid graphene-silicon waveguide. Consequently, the LGI will be significantly enhanced, and polarization-insensitive modulation can be achieved because of the tunable EA features of graphene. To demonstrate the use of the proposed hybrid graphene-silicon waveguide structure, we suggested and analyzed a novel polarization-insensitive EAM for on-chip communications. The width, thickness, and length of the proposed EAM are *W* = 600 nm, *H* = 300 nm, and *L*, respectively. When the input light is launched into this device, its propagating mode will experience different attenuation behaviors along the propagation direction depending on the modulation voltages applied on the graphene layers. The modal attenuation features are polarization-insensitive and the available optical bandwidth is relatively large. After the modulation, the modulated light output from this device can be leveraged directly for other integrated signal-processing components. Since the graphene tunable conductivity is the pivotal attribute to set a large difference in the mode power attenuation (MPA) for the proposed device, we will first study the modal features of the hybrid graphene-silicon waveguide and then apply it into the polarization-insensitive EAM to achieve better performance.

## 3. Optical Properties of Hybrid Graphene-Silicon Waveguide

The discovery of graphene has accelerated the development of on-chip photonic devices to be employed as high-performance building blocks for optical interconnects. Graphene can be analyzed theoretically using either the isotropic [32,33] or anisotropic [34,35] models. In the isotropic model, ultra-high ME can be easily obtained (e.g., a record high ME of ~4.5 dB/µm [33]) by insetting graphene layers into the slot region of the horizontal slot waveguide where its light field is greatly confined in the graphene layer region by the epsilon-near-zero effect [33]. However, such theoretical predictions cannot be achieved in experiments. More research revealed that we ought to treat graphene as an anisotropic material since it has only one single-atom layer; the carriers are strongly confined to move in this layer [36,37]. Therefore, only the in-plane permittivity and conductivity of graphene can be adjusted by changing its Fermi level (or chemical potential *µ*_c_). The corresponding out-of-plane permittivity remains unchanged at around 1 or 2.5 [37]. For the in-plane conductivity of graphene, an analytic expression can be deduced from the Kubo formula, which comprises intra- and inter-band contributions as follows [38]:

(1) $$\sigma_{\mathrm{intra-band}}=i\frac{e^{2}k_{B}T}{\pi \hbar^{2}(\omega +i\tau^{-1})}\left[\frac{\mu_{c}}{k_{B}T}+2\ln\left(e^{-\mu_{c}/(k_{B}T)}+1\right)\right],$$

and

(2) $$\sigma_{\mathrm{inter-band}}=\frac{ie^{2}(\omega +i\tau^{-1})}{\pi \hbar^{2}}\int_{0}^{\infty }\frac{f_{d}(-\varepsilon )-f_{d}(\varepsilon )}{{\left(\omega +i\tau^{-1}\right)}^{2}-4{\left(\varepsilon /\hbar \right)}^{2}}d\varepsilon ,$$

where *e* is the electron charge, *ħ* is the reduced Planck’s constant, *T* is the temperature, *k*_B_ is the Boltzmann constant, *τ* is the momentum relaxation time, *ω* is the angular frequency, *ε* is the energy, and $f_{d}(\varepsilon )={\left(e^{\left(\varepsilon -\mu_{c})/(k_{B}T\right)}+1\right)}^{-1}$ is the Fermi-Dirac distribution. If $k_{B}T\ll \left|\mu_{c}\right|,hv$ is satisfied, the inter-band conductivity can be estimated as [38]:

(3) $$\sigma_{\mathrm{inter-band}}=i\frac{e^{2}}{4\pi \hbar }\ln\left(\frac{2\left|\mu_{c}\right|-\hbar (\omega +i\tau^{-1})}{2\left|\mu_{c}\right|+\hbar (\omega +i\tau^{-1})}\right).$$

Here, we have set *τ* = 0.5 ps (based on a conservatively estimated carrier mobility of 10,000 cm^2^·V^−1^·s^−1^) and *T* = 300 K. Moreover, thin 3D volumes and 2D sheets are the commonly assumed models in simulation because considering the graphene flake as one-atom layer thick and modeling the graphene layer as a 2D sheet gives better consistency by comparing the experimental and simulation results [39]. Therefore, in the following analyses, we will model the graphene layers as typical anisotropic 2D sheets and combine them with the multilayer silicon nanowires to form the hybrid graphene-silicon waveguide.

**Figure 2:**
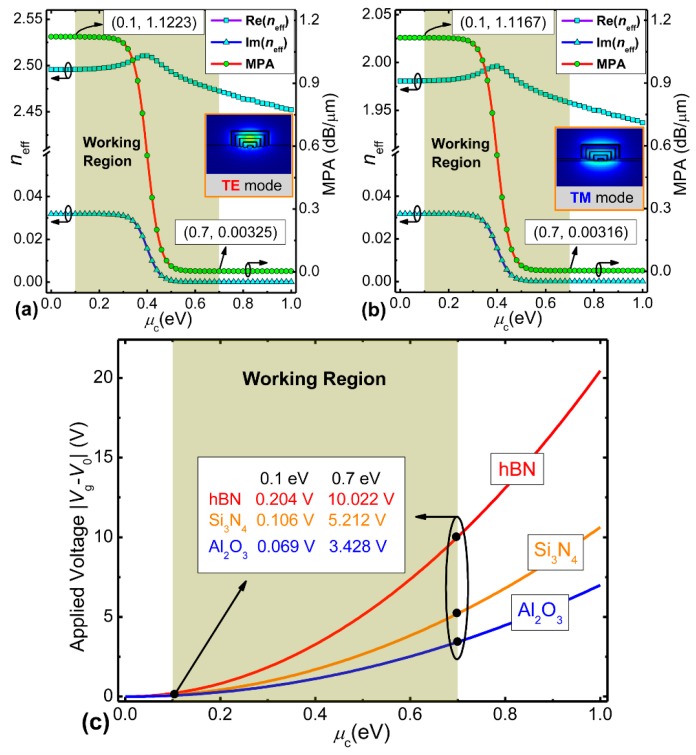
Modal properties of both polarization modes and applied voltages on the graphene flake. Real (Re(*n*_eff_)) and imaginary (Im(*n*_eff_)) parts of effective modal index of the hybrid graphene-silicon waveguide and the MPA versus chemical potential (*µ*_c_) of graphene for (**a**) transverse electric (TE) mode and (**b**) transverse magnetic (TM) mode. The insets show the electric field profiles (dominant component) at *µ*_c_ = 0.7 eV. (**c**) Applied voltage (|*V*_g_ − *V*_0_|) of the hybrid graphene-silicon waveguide as a function of the graphene chemical potential (*µ*_c_) for different spacer and insulating materials (Al_2_O_3_, Si_3_N_4_, hBN). The inset calculates the applied voltages for three different spacer and insulating materials at *µ*_c_ = 0.1 eV and 0.7 eV.

Figure 2a,b shows the effective modal index (real [Re(*n*_eff_)], imaginary [Im(*n*_eff_)]) of the hybrid graphene-silicon waveguide as a function of the graphene chemical potential (*µ*_c_) for both transverse electric (TE) and transverse magnetic (TM) modes at *λ* = 1.55 µm where the modal calculation is based on the above-mentioned surface conductivity model of graphene. The calculation engine is available from a commercial software package [40]. The widths and thicknesses of five inverted U-shaped graphene-silicon layers are *w*_1_ = 80 nm, *w*_2_ = 210 nm, *w*_3_ = 340 nm, *w*_4_ = 470 nm, *w*_5_ = 600 nm, and *h*_1_ = 40 nm, *h*_2_ = 95 nm, *h*_3_ = 150 nm, *h*_4_ = 205 nm, *h*_5_ = 260 nm, respectively. We note that when *µ*_c_ increases, Re(*n*_eff_) increases slightly first and then decreases quickly. The maximum value is located at *µ*_c_ = 0.4 eV, while Im(*n*_eff_) reveals a large change around *µ*_c_ = 0.4 eV. The reason is that if *µ*_c_ is under the half of the photon energy (*hv*/2 = 0.4 eV), inter-band transitions of graphene layers will dominate and generate a strong optical absorption, resulting in the high imaginary part Im(*n*_eff_). If *µ*_c_ > *hv*/2, inter-band transitions will be blocked owing to the Pauli blocking mechanism, and the corresponding graphene layers will become transparent [41,42]. To quantify this absorption manner, we used the parameter of MPA, which is derived from the modal effective index [27]:

(4) MPA=40πlog(e)Im(neff)/λ,

Figure 2a,b shows that MPA presents a huge variation at around *µ*_c_ = 0.4 eV and gradually remains constant when *µ*_c_ deviates from 0.4 eV. To render the proposed EAM to have a relatively large tolerance for *µ*_c_, we set *µ*_c_ = 0.1 eV and *µ*_c_ = 0.7 eV as the “OFF” and “ON” states, corresponding to the MPA of ~1.1223 dB/µm (1.1167 dB/µm) and ~0.0033 dB/µm (0.0032 dB/µm) for the TE (TM) mode, respectively. Figure 2a,b also depicts the electric field profiles at the “ON” state for both polarizations, while those at the “OFF” state are not shown, since their field profiles are very similar. Considering the anisotropic property of the ultra-thin graphene layer, only the graphene layers located in the horizontal plane can contribute to the EA modulation under the TE polarization, while the slot effect generated by different refractive indices between adjacent materials (hBN and Si) is on the vertical plane. Thus, the vertical slot effect could hardly affect the horizontal in-plane modulation performance due to their orthogonal polarization states. Similar behaviors can also be observed for the TM polarization. By applying suitable voltages on the graphene layers through metal contacts on both sides of the central hybrid graphene-silicon waveguide, the *µ*_c_ of the graphene will vary correspondingly, and the relation between them can be calculated via the parallel plate capacitor model as follows [43]:

(5) $$\mu_{c}=\hbar V_{F}\sqrt{\frac{\pi \varepsilon_{0}\varepsilon_{d}\left|V_{g}-V_{0}\right|}{h_{d}e}},$$

where *V*_F_ is the Fermi velocity (≈10^6^ m/s), *ε*_0_ is the permittivity of vacuum, *ε*_d_ and *h*_d_ are the relative dielectric constant and thickness of the spacer layer between two graphene layers, respectively, and *V*_0_ denotes the bias voltage generated by the natural doping. Here, we treat |*V*_g_ − *V*_0_| as the applied voltage for simplicity, and |*V*_g_ − *V*_0_| versus *µ*_c_ for different spacer and insulating materials (Al_2_O_3_, Si_3_N_4_, hBN) is shown in Figure 2c where the spacer or insulating thickness *h*_d_ is 6 nm. The inset calculates the applied voltages at the “OFF” and “ON” states for different spacer materials. Among these typical spacer and insulating materials, hBN is the best choice, since hBN encapsulation of graphene layers can maintain graphene’s optical and electrical properties, e.g., a high carrier mobility (~140,000 cm^2^·V^−1^·s^−1^) [30], which is important to realize high-speed EAM. Besides, with the relatively low dielectric constant of hBN (~3.9), the electrical resistance-capacitance (*RC*) constant is reduced, which increases the 3 dB modulation bandwidth of the hybrid graphene-silicon-based polarization-insensitive EAM and reduces its energy consumption to some extent [44].

## 4. Results and Discussion

**Figure 3:**
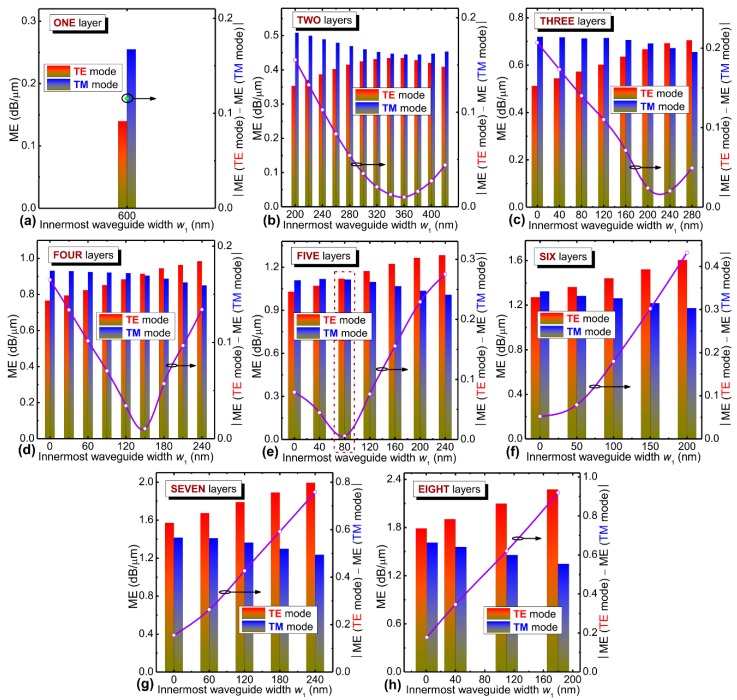
Modulation efficiencies (MEs) dependent on the dimensions and layer numbers of the graphene-silicon waveguide layer. The calculated MEs and their absolute difference for both polarizations as a function of the innermost waveguide width *w*_1_ of the hybrid graphene-silicon waveguide for different number of layers: (**a**) one layer, (**b**) two layers, (**c**) three layers, (**d**) four layers, (**e**) five layer, (**f**) six layers, (**g**) seven layers, and (**h**) eight layers, where a width relation of Δ*w*·(*N* − 1) + *w*_1_ = *W* is also applied.

To realize polarization-insensitive modulation with high ME and ultra-broad bandwidth, we designed a hybrid graphene-silicon waveguide using multi-deposited and multi-transferred methods, which can make the light interaction with graphene layers in its waveguide interior instead of in the cladding region with weak evanescent waves. Therefore, the dimensions of each of the inverted U-shaped graphene-silicon waveguide layer and the number of layers (*N*) are the key parameters. Here, the width-thickness ratio of every inverted U-shaped graphene-silicon waveguide layer was kept the same with that of the input/output silicon nanowire (*W* = 600 nm, *H* = 300 nm), and the width difference (Δ*w*) between adjacent layers was also kept the same for simplicity. Figure 3 shows the calculated MEs and their absolute difference for both polarizations as a function of the innermost waveguide width *w*_1_ of the hybrid graphene-silicon waveguide with one to eight layers, where a width relation of Δ*w*·(*N* − 1) + *w*_1_ = *W* applies based on the assumptions above, and ME = MPA (*µ*_c_ = 0.1 eV) − MPA (*µ*_c_ = 0.7 eV) [29]. We note that when the layer number *N* increases, ME gradually increases for both polarizations. On the other hand, when *N* increases, the minimum value of the absolute ME difference between the TE and TM modes decreases first and then increases, with the lowest value attained at *N* = 5 and *w*_1_ = 80 nm, which corresponds to the lowest polarization-dependence of the ME (<0.006 dB/µm). The obtained ME is relatively high for both polarizations (~1.11 dB/µm). When *N* > 5, the required fabrication process will become increasingly complex and the polarization-dependence of ME obviously increases when compared with that of *N* < 5. Therefore, *N* = 5 is our optimal choice, and the corresponding *w*_1_ and Δ*w* are 80 nm and 130 nm, respectively. Other waveguide widths (*w*_2_~*w*_5_) and thicknesses (*h*_1_~*h*_5_) were obtained using the relationship mentioned above.

**Figure 4:**
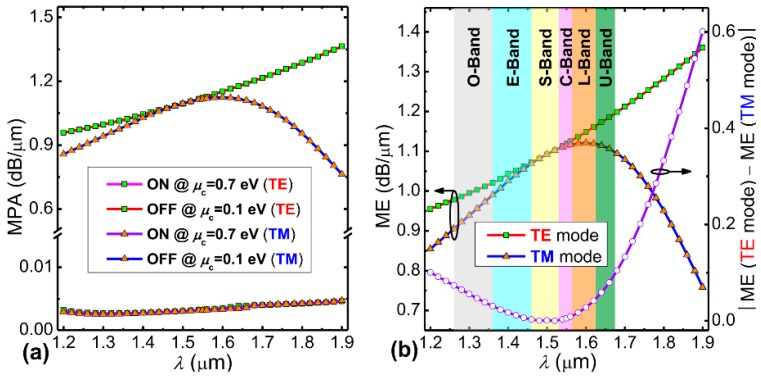
(**a**) The wavelength-dependence of MPA at both the “ON” and “OFF” states for TE and TM modes. (**b**) The corresponding wavelength-dependence of MEs and their absolute difference of the hybrid graphene-silicon waveguide for both polarizations.

**Figure 5:**
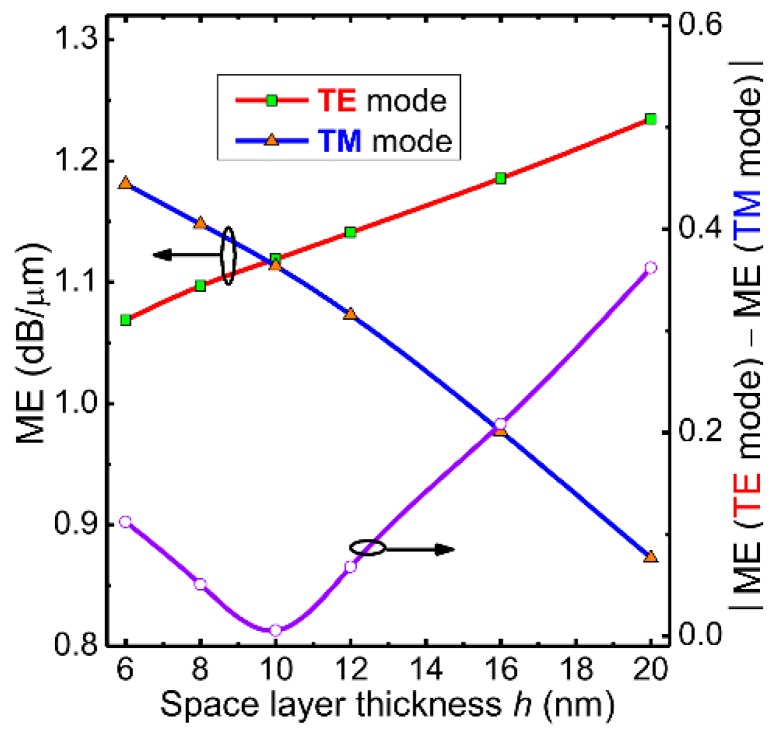
The calculated MEs and their absolute difference of the hybrid graphene-silicon waveguide for both polarizations versus the spacer layer thickness *h*.

In the following, by leveraging the zero bandgap (or broadband absorption) property of graphene [18,19], we improve the optical bandwidth of the proposed EAM to cover the main optical communication bandwidth. Figure 4a shows the variation of MPA at both the “ON” and “OFF” states for the TE and TM modes in the wavelength range from 1.2 to 1.9 µm based on the modal analysis with material dispersion considered [45]. At the “ON” state, both polarization modes show similar wavelength dependence. At the “OFF” state, the wavelength dependences of the two polarization states behave differently. When the wavelength increases, the MPA of TE mode gradually increases, but that of TM mode first increases and then decreases. To better illustrate this wavelength-dependence of the modulation feature, Figure 4b shows the MEs and their absolute difference for both polarizations. The wavelength ranges from O to U band are also marked. From Figure 4b, the MEs for both polarizations are almost equal in the S and C bands, corresponding to polarization-independent operation. If the ME discrepancy for both polarizations is set at <0.1 dB/µm, the available bandwidth is from 1.2 to 1.68 µm, which covers the full band of optical communications, giving rise to broadband EA modulation. We then studied the modulation performance of the proposed hybrid graphene-silicon waveguide as a function of the spacer layer (hBN) thickness *h* for both polarizations. Figure 5 shows the calculated MEs and their absolute difference. From Figure 5, the ME curves of the TE and TM modes exhibit an opposite variation trend. The two curves intercept near *h* = 10 nm, which corresponds to the polarization-insensitive modulation.

**Figure 6:**
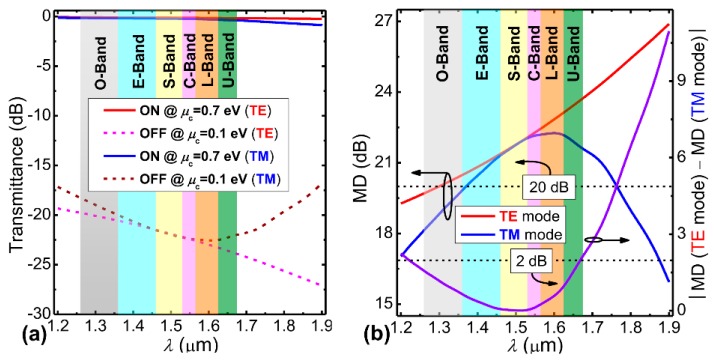
Transmission spectra and the derived wavelength-dependence of the modulation depths. (**a**) Transmission spectra of the proposed EAM at the “ON” and “OFF” states for both polarizations. The length of the EAM is 20 µm. (**b**) The corresponding modulation depths (MDs) and their absolute difference for both polarizations versus the same wavelength range. The two horizontal lines represent the MD of 20 dB and MD discrepancy of 2 dB, respectively.

Based on the obtained high ME, ultra-broadband, and polarization-insensitive properties, we further propose a hybrid graphene-silicon-based polarization-insensitive EAM with a modulation length of only *L* = 20 µm. We analyzed its transmission properties by using a three dimensional-finite difference time domain (3D-FDTD) method [40]. Figure 6a shows the transmission spectra of the proposed EAM at the “ON” and “OFF” states for both polarizations, where the simulation wavelength spectrum is from 1.2 to 1.9 µm and the material dispersion is included based on Palik’s book [45]. It is noteworthy that the difference of optical transmittances between the “ON” and “OFF” states is very close for the TE and TM modes within the full band of optical communications, especially for the S and C bands. To better show this phenomenon, we also plotted MDs and their absolute difference for both polarizations as a function of the same wavelength, as shown in Figure 6b, where MD = transmittance (*µ*_c_ = 0.7 eV) − transmittance (*µ*_c_ = 0.1 eV) [29]. The wavelength-dependence of the MD curves is very similar to that of the ME curves for both polarizations shown in Figure 4b based on the modal analysis, which also demonstrates the reliability of the 3D-FDTD method. If we use the criterion of the MD discrepancy being lower than 2 dB, the allowable bandwidth is from 1.221 to 1.668 µm, which is slightly smaller than that based on the result of modal analysis, since the input/output silicon nanowire will incur weak coupling loss in the transmission. Moreover, to make the proposed hybrid graphene-silicon-based EAM operate at high MD, we set another criterion, i.e., MD > 20 dB, and the corresponding allowable bandwidth is from 1.367 to 1.771 µm. By considering these two criteria, the final available bandwidth is 301 nm (from 1.367 to 1.668 µm), covering the whole S, C, L bands and the most of E and U bands, where ILs and back reflection losses of both polarizations are lower than 0.4 dB and −27.8 dB, respectively, within this range.

**Figure 7:**
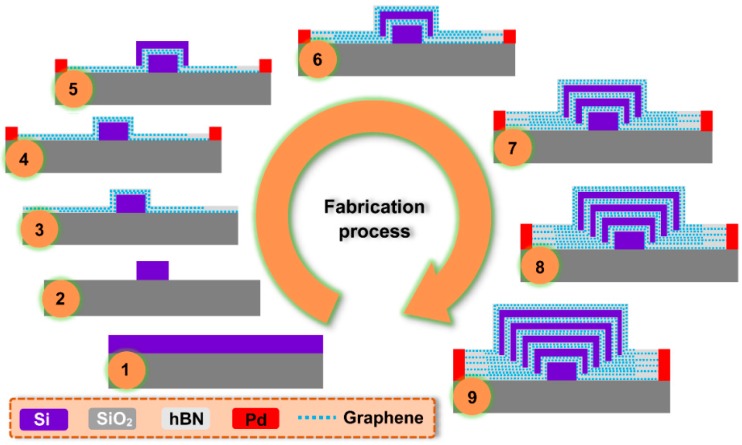
Fabrication process of the proposed hybrid graphene-silicon-based polarization-insensitive EAM. Starting from a silicon-on-insulator (SOI) wafer (step 1) and then etching to form the bottom silicon nanowire (step 2). The hBN-graphene-hBN-graphene-hBN heterostructure is further deposited on the bottom silicon nanowire (step 3), and the effective metal contacts on both sides of the waveguide are also done (step 4). The second silicon nanowire is grown on the first hybrid graphene-silicon waveguide layer (step 5), and the hBN-graphene-hBN-graphene-hBN heterostructure is also deposited, including the metal contacts (step 6). Using the same method to fabricate the third to the fifth hybrid graphene-silicon waveguide layers consecutively (step 7 to step 9). Note that for clarity, the silicon substrate of the SOI wafer is not shown.

To fabricate the proposed EAM, an SOI wafer with a 40-nm thick top silicon layer and a 2 µm thick buried oxide layer was employed. The detailed fabrication process can be found in Figure 7. For the first hybrid graphene-silicon waveguide layer, the bottom silicon nanowire with a width of *w*_1_ = 80 nm and a length of *L* = 20 µm was patterned with an E-beam lithography and etched to the buried oxide layer with an inductively coupled plasma reactive ion etching process (step 1 and step 2). Then, two graphene layers grown by CVD were transferred onto the surface of the bottom silicon nanowire, including the spacer layer of hBN [46,47], where the hBN encapsulation of two graphene layers (hBN-graphene-hBN-graphene-hBN heterostructure) was fabricated by employing multi-level stacking of the 2D material with a van der Waals assembly process (step 3) [30,48]. The thickness of the spacer layer between the two graphene layers was 6 nm. In this process, the spacer layer including its encapsulated graphene layers was extended on both sides of the central hybrid graphene-silicon waveguide to ensure effective metal contacts (step 4) [49,50]. The lateral distance between the metal contact and the central waveguide was set as 1.5 μm on each side to prevent metal absorption. For the second hybrid graphene-silicon waveguide layer, the second silicon nanowire was grown on the first hybrid graphene-silicon waveguide layer by epitaxy technology and then patterned and etched to form the required waveguide dimension (*w*_2_ = 210 nm, *h*_2_ = 95 nm, and *L* = 20 µm) (step 5). The procedures of fabrication and transferring of graphene layers and spacer layers are similar with those described in the first hybrid graphene-silicon waveguide layer, including the metal contacts on both sides (step 6). We then repeated this process three more times to fabricate the third to the fifth hybrid graphene-silicon waveguide layers consecutively (step 7 to step 9). The corresponding width and thickness of the silicon nanowire were *w*_3_ = 340 nm, *w*_4_ = 470 nm, *w*_5_ = 600 nm, and *h*_3_ = 150 nm, *h*_4_ = 205 nm, *h*_5_ = 260 nm, respectively. The length was *L* = 20 µm. Finally, the input and output silicon nanowires (*W* = 600 nm, *H* = 300 nm, length = 5 µm) were also fabricated on the same wafer to be butt-coupled with the hybrid graphene-silicon based EAM, allowing for easy and direct connection with other integrated components. Finally, by applying switching voltages on the graphene layers using fabricated metal electrodes, the input light could be modulated by this highly-efficient, ultra-broadband, and polarization-insensitive EAM.

**Figure 8:**
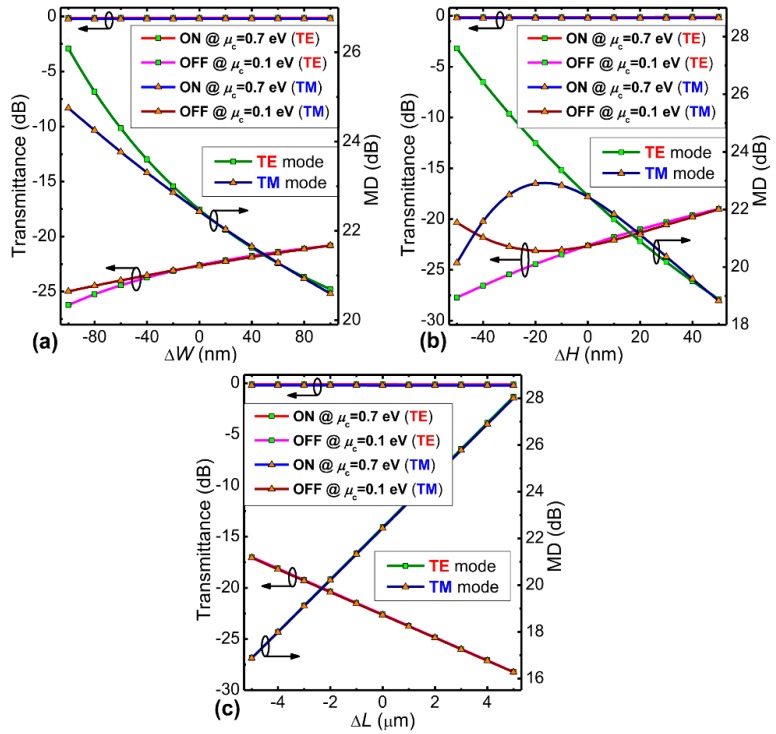
Fabrication tolerance analyses. Transmittance and MD of the proposed modulator as its total (**a**) width *W*, (**b**) thickness *H*, and (**c**) length *L* deviate from their designed values at the “ON” and “OFF” states for both polarizations, where the size deviations of waveguide width and thickness are assumed to be evenly distributed in every hybrid graphene-silicon waveguide layer.

**Figure 9:**
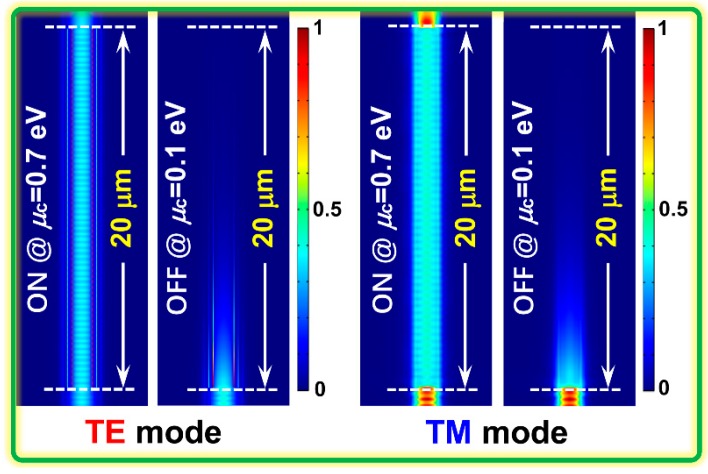
Evolution of the optical field intensity of the input TE and TM modes along the propagation distance (*L* = 20 µm) through the designed hybrid graphene-silicon-based polarization-insensitive EAM at the “ON” and “OFF” states.

Next, to investigate the performance deterioration brought by fabrication imperfections in practice, we analyzed the fabrication tolerances of the width *W*, thickness *H*, and length *L* of the hybrid graphene-silicon-based EAM. The deviations of *W* and *H* were assumed to be evenly distributed in every hybrid graphene-silicon waveguide layer based on the same fabrication processing flow. Figure 8 shows the transmittance and MD of the devices as its *W*, *H*, and *L* deviate from their designed values (*W* = 600 nm, *H* = 300 nm, *L* = 20 µm) at the “ON” and “OFF” states for both polarizations. When *W* and *H* increase, MD gradually decreases for both polarizations owing to the increase in mode field area and weakened LGI, except for the TM mode in the range of Δ*H* = [−50 nm, −10 nm] where the MD of the TM mode has a peak value at Δ*H* = −20 nm. Moreover, the polarization-insensitive property of the proposed EAM remains unaffected if ΔW and ΔH are kept within the ranges of [0, 100 nm] and [0, 50 nm], respectively, which gives a relatively large tolerance for fabrication. For the device length *L*, the variations of transmittance and MD of the TE and TM modes are nearly the same, showing excellent polarization-insensitive properties. In addition, a higher MD for this device can be achieved only by increasing the device length because its insertion loss is very low and increases slowly owing to the small absorption loss at the “ON” state for both polarizations. Finally, Figure 9 plots the evolution of the optical field intensity for both polarizations along the optimized hybrid graphene-silicon-based polarization-insensitive EAM at both the “ON” and “OFF” states. The structural dimensions are based on the optimized values above, and the operating wavelength is 1.55 µm. When the EAM operates at the “ON” state, the input TE and TM modes will pass through the modulation region with very low loss. In contrast, when the EAM operates at the “OFF” state, the input TE and TM modes will attenuate quickly as they enter into the modulator, which is owed to the high absorption loss of graphene layers.

Besides the above-analyzed optical properties of the proposed hybrid graphene-silicon-based polarization-insensitive EAM, its corresponding electrical characteristics are also critical, such as the 3 dB modulation bandwidth (*f*_3dB_) and the energy consumption (*E*), which are associated with the total capacitance (*C*) and resistance (*R*) of the optical device, *f*_3dB_ = 1/(2*πRC*), *E* = *CV*^2^/4 (*V* stands for the switching voltage between “OFF” and “ON” states) [40,42]. Here, we estimated the electrical characteristics of the proposed EAM using the parallel plate capacitor and the equivalent electrical circuit model for simplicity [27,28,29]. By considering the multilayer hybrid graphene-silicon waveguide structure and the overlapping region between graphene layers, the total resistance of the device is estimated to be ~80 Ω (including the graphene sheet resistance and the metal-graphene contact resistance) and the total capacitance (*C*) is estimated to be ~326 fF. Thus, *f*_3dB_ and *E* are derived to be ~6.1 GHz and ~7.8 pJ/bit, respectively. To further enhance the electrical properties of the proposed EAM, its total resistance and capacitance should be effectively reduced, especially for the capacitance, which can be realized by cutting down the overlapping area between two corresponding graphene layers, choosing the spacer/insulating layer with the lower dielectric constant, and increasing the thickness of the spacer/insulating layer. In addition, traveling-wave electrode designs could also be leveraged to enhance the modulation performance of the proposed EAM [51].

## 5. Conclusions

In summary, using multi-deposited and multi-transferred methods, we propose a highly-efficient, ultra-broadband, and polarization-insensitive EAM, the key part of which is a hybrid graphene-silicon waveguide formed by five inverted U-shaped graphene-silicon layers. In order to effectively enhance LGI, multi-deposited and multi-transferred graphene-silicon layers are employed such that light interacts with the graphene layers at high optical intensity in the interior of the hybrid graphene-silicon waveguide rather than at weak optical intensity in the cladding region, as commonly done. We then optimized the dimensions of each hybrid graphene-silicon layer based on the inverted U-shaped waveguide structure and achieve an ME of ~1.11 dB/µm and an ME discrepancy of lower than 0.006 dB/µm for both polarizations, which are significant improvements over previous results. By using this structure, we designed and analyzed a hybrid graphene-silicon-based polarization-insensitive EAM only 20 µm long. The MD and IL were calculated to be > 22 dB and < 0.23 dB at 1.55 µm, respectively, and the available bandwidth can be over 300 nm (from 1.367 to 1.668 µm) by simultaneously keeping MD greater than 20 dB and MD discrepancy less than 2 dB. The MD of the designed EAM can be further improved by increasing its length. The electrical properties of the EAM (e.g., 3 dB modulation bandwidth and energy consumption) were studied. Several methods were also proposed to enhance its electrical features. With high ME, ultra-broad bandwidth, polarization-insensitivity, compact footprint, and scalable performance, the proposed hybrid graphene-silicon-based EAM will find applications in the high performance hybrid silicon modulators for high capacity on-chip optical interconnects.
